# Reemergence of human malaria in Atlantic Forest of Rio Grande do Sul, Brazil

**DOI:** 10.1590/0074-02760210064

**Published:** 2021-07-12

**Authors:** Alessandra Bittencourt de Lemos, Onilda Santos da Silva, Sandra Cristina Deboni, Valdir Schallemberger, Edmilson dos Santos, Marco Antônio Barreto de Almeida, Anne Andrea Dockhorn Marth, Sidnei Silva, Aline Rosa de Lavigne Mello, Teresa Fernandes Silva-do-Nascimento, Maria de Fátima Ferreira-da-Cruz, Ricardo Lourenço-de-Oliveira, Jáder da Cruz Cardoso

**Affiliations:** 1 Universidade Federal do Rio Grande do Sul, Departamento de Microbiologia, Imunologia e Parasitologia, Porto Alegre, RS, Brasil; 2 Secretaria da Saúde do Estado do Rio Grande do Sul, Centro Estadual de Vigilância em Saúde, Divisão de Vigilância Epidemiológica, Porto Alegre, RS, Brasil; 3 Secretaria da Saúde do Estado do Rio Grande do Sul, Centro Estadual de Vigilância em Saúde, Laboratório Central de Saúde Pública, Porto Alegre, RS, Brasil; 4 Secretaria da Saúde do Estado do Rio Grande do Sul, Centro Estadual de Vigilância em Saúde, Divisão de Vigilância Ambiental em Saúde, Porto Alegre, RS, Brasil; 5 Secretaria da Saúde do Estado do Rio Grande do Sul, 18ª Coordenadoria Regional de Saúde, Osório, RS, Brasil; 6 Fundação Oswaldo Cruz-Fiocruz, Instituto Nacional de Infectologia Evandro Chagas, Laboratório de Parasitologia, Rio de Janeiro, RJ, Brasil; 7 Fundação Oswaldo Cruz-Fiocruz, Instituto Oswaldo Cruz, Laboratório de Pesquisas em Malária, Rio de Janeiro, RJ, Brasil; 8 Fundação Oswaldo Cruz-Fiocruz, Instituto Oswaldo Cruz, Laboratório de Mosquitos Transmissores de Hematozoários, Rio de Janeiro, RJ, Brasil

**Keywords:** Anopheles, Plasmodium vivax, Plasmodium simium, malaria, Kerteszia

## Abstract

Unforeseen *Plasmodium* infections in the Atlantic Forest of Brazilian Extra-Amazonian region could jeopardise malaria elimination. A human malaria case was registered in Três Forquilhas, in the Atlantic Forest biome of Rio Grande do Sul, after a 45 years’ time-lapsed without any malaria autochthonous notification in this southern Brazilian state. This finding represents the expansion of the malaria distribution areas in Brazil and the southernmost human malaria case record in South America in this decade. The coexistence of the bromeliad-breeding vector *Anopheles* (*Kerteszia) cruzii* and non-human primates in the Atlantic Forest regularly visited by the patient claimed for the zoonotic origin of this infection. The reemergence of Atlantic Forest human malaria in Rio Grande do Sul was also discussed.

Malaria is an acute febrile infectious disease caused by *Plasmodium* parasites transmitted by infecting bites of anopheline female mosquitoes. Malaria affects thousands of people worldwide and, therefore, represents a serious public health problem, especially in populations of tropical and subtropical regions of Africa, Asia and Central and South Americas.^1^ In 2019, about 89% of malaria cases registered in Brazil were caused by *Plasmodium vivax*, while *P. falciparum* accounted for just over 10% of cases, and *P. malariae* was rarely diagnosed.^2^ In Brazil, there are two main epidemiological and transmission profiles: (i) the Amazon basin rainforest malaria, which accounts for more than 99% of the reported malaria cases and (ii) Atlantic Forest malaria that, despite accounting for only 0.08% of cases, complicates control measures and represents an important public health problem because of the case-fatality rate, due to the increased time elapsed for the diagnosis.^2^ ^,^ ^3^ Differently from the Amazonian Region, where *Anopheles (Nyssorhynchus) darlingi* is responsible for the bulk of the endemic and epidemic transmission, *Anopheles (Kerteszia) cruzii* has been considered the main vector in the Atlantic Forest, a biome rich in bromeliads, the larval habitat of *Kerteszia* mosquitoes.^4^ Coincidently, *An. cruzii* is also the main vector of simian malaria in Atlantic Forest areas.^4^ ^,^ ^5^ In certain circumstances, the bites of *An. cruzii* may be numerous both at the tree canopies and on ground level, and a high prevalence of both human and simian malaria would be expected.^4^ ^,^ ^6^ ^,^ ^7^ ^,^ ^8^

Rio Grande do Sul is the southernmost Brazilian State. The first records of malaria in this state date back to 1900 in a port city named Rio Grande, located in the southern state coast.^9^ In 1918, the disease was recorded at the northern coast, in the municipality of Torres, bordering the State of Santa Catarina.^10^ ^,^ ^11^ Thereafter, the northern coastal zone between the municipalities of Torres and Osório, essentially from latitude 29º30’ to 29º59’, was considered the hot spot malaria transmission area until the end of the 1960s. After several control actions, autochthonous human malaria was no longer recorded in Rio Grande do Sul.^12^ ^,^ ^13^ ^,^ ^14^ ^,^ ^15^ ^,^ ^16^ Then, after more than four decades, an unexpected malaria case was detected in April 2014, in a 58-year-old white male, diabetic insulin dependent, living in the anopheline-free urban area of Terra de Areia (29º35’13’’S 50º03’59’’W), a municipality at the northern coastal zone distant 45 km from Torres and 122 km from Porto Alegre, the capital of Rio Grande do Sul (Fig. 1). Even though the patient lives in an urban area, he often spent the weekends and holidays in his vacation home at the rural area of the Três Forquilhas municipality (site 1 - 29º22’56.1”S 50º10’03.6”W) and hunting in the vicinity woods of his employee’s lands (site 2 - 29º23’59.43”S 50º07’54.46”W) (Fig. 1). Both 1 and 2 sites are located in the Atlantic Forest biome (Fig. 2). At the onset of the symptoms, the patient reported cough, runny nose and body aches on April 24th. After seven days, symptoms progressed to fever, sweating, chills, dyspnea and intense tremors. On May 11, and after 10 days of treatment for a suspected leptospirosis, the patient was admitted to the Intensive Care Unit of São Lucas Hospital in Porto Alegre, where his clinical features progressed to renal failure, thrombocytopenia and disorientation. Promptly, the blood film examination detected the presence of *Plasmodium* and the protocol treatment for severe and complicated malaria, recommended by the Brazilian Ministry of Health, was started.^17^ The next day, after notification to the Epidemiological Surveillance of Porto Alegre municipality, thin blood smears were forwarded to the Parasitology Laboratory of the Central Laboratory of the State Department of Health of Rio Grande do Sul - LACEN/SES-RS, where *P. vivax* malaria infection was diagnosed with a parasitaemia of 5,000 parasites/μL. The patient recovered and left the hospital on May 20th.

**Fig. 1:**
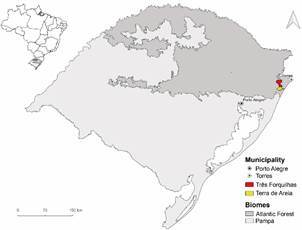
Rio Grande do Sul State, highlighting the Três Forquilhas and Terra de Areia municipalities, in the Atlantic Forest Biome.

**Fig. 2:**
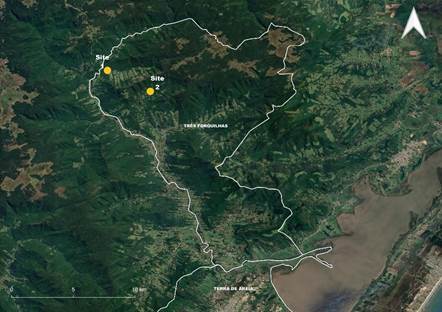
location of the areas visited by the malaria patient in forest environments of the municipality of Três Forquilhas. Site 1) Patient vacation home; Site 2) Patient’s employee lands.

Concomitantly, according to the recommendations of the Brazilian Ministry of Health, a patient blood sample taken during hospitalisation was sent to the Extra-Amazon Reference Centre for Malaria Diagnosis, at Fundação Oswaldo Cruz, for confirmation of malaria diagnosis by parasitological and molecular methodologies and to rule out the possibility of co-infection by polymerase chain reaction (PCR). For this end, the genomic DNA was extracted from 1 mL whole blood using QIAamp midi columns, as described by the manufacturer (Qiagen^®^). *P. simium/P. vivax* was diagnosed by conventional^18^ and real-time^19^ PCRs, and no *P. falciparum* or *P. malariae* infection was detected;^20^ ^,^ ^21^ the same was true for microscopic examination.

To identify vectors possibly involved in the transmission, in November 17 and 19, 2014, a team from the Environmental Health Surveillance (CEVS) collected mosquitoes in site 2, (Fig. 3) using Shannon trap between 6:30 - 9:30PM. Mosquitoes were sent to the Laboratory of Mosquitoes and Hematozoa Transmitters (LATHEMA) of Fundação Oswaldo Cruz and 268 specimens of *Anopheles* (*Kerteszia*) *cruzii* were identified. Aiming to identify *Plasmodium* infection in these samples, entire bodies of mosquitoes were polled (≤ 5 individuals each) and homogenised in 50 µL of DNA-free sterile distilled water by using the Precellys 24^®^ tissue homogeniser in bead tubes. DNAzol (150 µL) was added to the homogenates, following centrifugation (9600 x g, 10 min). DNA was extracted from 160 µL of supernatant after precipitation with 100 µL of 100% ethanol, following centrifugation (9600 x *g*, 10 min). The pellet was resuspended with 160 µL of 70% ethanol and subsequently centrifuged as above. After evaporation at room temperature, the pellet was finally resuspended in 40 µL of DNA-free sterile distilled water and incubated at 4ºC overnight. Then, PCR was performed employing the same protocols used for detection of the human malaria infection. All mosquitoes tested negative for *P. falciparum*, *P. vivax* and *P. malariae* infections.

**Fig. 3:**
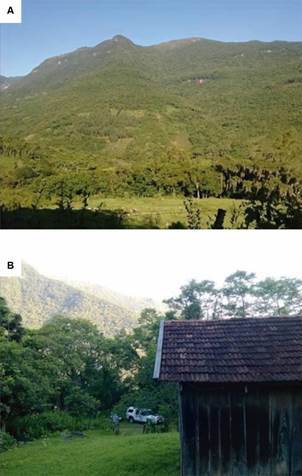
probable infection site, in the municipality of Três Forquilhas. (A) Landscape profile of the region; (B) Patient employee’s lands.

The epidemiological investigation did not detect any introduced or imported malaria case either in the municipality where the patient lives (Terra de Areia, an urban area) or in the municipality regularly visited by the patient (Três Forquilhas). In addition, the patient did not report any travel to Brazilian or foreign malaria endemic areas and did not receive a blood transfusion. All these data, plus the capture of *An. cruzii* in Três Forquilhas, as well as the presence of howler monkeys, the main reservoir of malaria parasites-infecting humans^5^ ^,^ ^7^ ^,^ ^22^ ^,^ ^23^ in Atlantic Forest sites visited by the patient, strongly points to Três Forquilhas as the infection site and raised the hypothesis of a zoonotic malaria. Indeed, simian parasites originating tertian malaria human outbreaks have been reported in Atlantic Forest of Southeast Brazil^23^ and, in the same way, the vast majority of malaria cases consisted of people living in urban areas who went to Atlantic Forest for leisure. It is well known that two simian *Plasmodium* species infecting-humans occur in South America: *P. simium* and *P. brasilianum. P. brasilianum* and *P. malariae* are considered the same species^24^ and the parasite is conventionally called *P. brasilianum* when detected in monkeys and *P. malariae* when diagnosed in humans. *P. simium* has a high degree of morphological, genetic, and immunologic similarities with *P. vivax* ^25^ ^,^ ^26^ and, therefore, it is possible to detect this parasite using in house *P. vivax* PCRs, as used in this work. While *P. brasilianum* is spread in the South America continent and has been found infecting several non-human primate species, *P. simium* has only been found in southern and southeastern Brazilian Atlantic Forest biome and infecting few non-human primate species.^7^ ^,^ ^22^ ^,^ ^27^ ^,^ ^28^ ^,^ ^29^ In fact, in 1969, when Rio Grande do Sul was already considered a human malaria-free area, *P. simium* was detected in *Alouatta guariba clamitans* (red-howler monkey) from the metropolitan region of Porto Alegre.^30^ More recently, *P. simium* has also been recorded in the same non-human primate species from two other Rio Grande do Sul municipalities: in 2003, in a blood sample of a free-living ill animal at the State Park of Itapuã, in the municipality of Viamão^31^ and in 2017, in captive animals, from the municipality of Morro Reuter,^32^ respectively, 135 to 90 km away from Três Forquilhas where the malaria case here reported occurred. Interesting, the only anopheline species found in the sites visited by the patient was *An. cruzii*, a neotropical, exophilic and opportunistic blood feed species that may bite non-human primate at the canopy of trees, and humans at the ground level in the forest and surroundings capable to transmit, therefore, zoonotic malaria of simian origin.^4^ ^,^ ^5^ ^,^ ^33^ This mosquito vector breeds in water hold in bromeliads and thus, the malaria it disseminates is called “bromeliad malaria” and is usually transmitted outdoors to people working or living in close contact or visiting this ambient for leisure activities,^7^ ^,^ ^23^ as happened with the Rio Grande do Sul malaria case here reported.

Because of the lack of detection of human malaria throughout Rio Grande do Sul for more than four decades, the malaria case here reported was much probably detected only due its severity requiring hospitalisation. Since no *P. falciparum* co-infection was disclosed, this unusual severity clinical manifestation could be related to pre-existing comorbidity (diabetes) associated with the delay of the diagnosis, since the signs and symptoms of human malaria infections originating from non-human primates in the Atlantic Forest are usually mild to moderate.^34^

The malaria case here reported showed the reemergence of human malaria in the southernmost Brazil as well as in South America as a whole, since countries with territories of similar or lower latitudes (Uruguay, Chile and Argentina) have been considered malaria free areas for two decades now.^35^

In Rio Grande do Sul, as in other Brazilian areas of extra-Amazonia, the large-time gap with no malaria case detection certainly contribute to rule out the possibility of malaria infection in febrile patients. Therefore, health units of the Rio Grande do Sul municipalities surrounding Atlantic Forest biome should include malaria as a differential diagnosis, and surveillance network of these regions must be attentive to febrile patients seeking care at health centers, aiming to improve the diagnosis and timely treatment cases. Finally, studies on the natural infection of *Plasmodium* species in non-human primates and *Anopheles* (*Kerteszia*) mosquitoes as well as serological surveys should be performed as eco-epidemiological surveillance strategies for identification of Atlantic Forest biome malaria transmission sites in Southern Brazil.
